# 6 and 12 month outcomes in patients following COVID-19-related hospitalization: a prospective monocentric study

**DOI:** 10.1007/s11739-022-02979-x

**Published:** 2022-04-09

**Authors:** Giuseppe Pio Martino, Devis Benfaremo, Giuseppina Bitti, Gianluca Valeri, Laura Postacchini, Annalisa Marchetti, Stefano Angelici, Gianluca Moroncini

**Affiliations:** 1 UOC Medicina Interna Fermo, ASUR Marche, Area Vasta 4, Ancona, Italy; 2 Clinica Medica, Dipartimento Di Medicina Interna, AOU Ospedali Riuniti Di Ancona, Via Conca 71, 60126 Ancona, Italy; 3 UOC Radiodiagnostica, ASUR Marche, Area Vasta 4, Ancona, Italy

**Keywords:** COVID-19, Post-acute COVID-19 syndrome, Complications

## Abstract

The long-term consequences of COVID-19 in those who recover from acute infection requiring hospitalization have not been defined yet. In this study, we aim to describe the long-term symptoms and respiratory outcomes over 12 months in patients hospitalized for severe COVID-19. In this prospective cohort study, patients admitted to hospital for severe COVID-19 were prospectively followed up at 6 and 12 months after discharge from the Hospital of Fermo, Italy. Patients were interviewed for persisting symptoms and underwent physical examination, routine blood test, pulmonary function tests, chest high-resolution CT (HRCT), and 6 min walking test. A total of 64 patients were evaluated and participated in this study. The mean age of participants was 68 years, 41 (64%) were males, and the median body mass index (BMI) was 26 kg/m^2^. After 6 months, 36% of patients reported persistent dyspnea, 37.5% persistent fatigue, 30.6% hair loss, 14% arthralgia and 11% memory and attention deficits. The rate of these symptoms reduced at the 12 month follow-up. At least 50% of the patients reported anxiety and depression symptoms. At 6 months 57.4% of patients showed reduced DLCO and 21.3% reduced FVC% and improvement at 12 months was noted for FVC but not for DLCO and TLC. Persistent radiographic abnormalities, most commonly ground-glass opacities and interstitial changes, were observed at both timepoints in many patients. Long-term symptoms and pulmonary deficits are common in patients admitted for severe COVID-19. Further studies are needed to assess the clinical significance of long-term consequences of severe COVID-19.

## Introduction

Long COVID or post-acute CoronaVirus Disease 19 (COVID-19) syndrome is a recently described condition characterized by persisting symptoms after acute Severe Acute Respiratory Syndrome—CoronaVirus 2 (SARS-CoV-2) infection that cannot be explained by an alternative diagnosis [1].

A variety of symptoms or clinical sequelae after COVID-19 have been reported. Most patients report annoying symptoms such as fatigue, dyspnea, cognitive impairment, sleep disturbances, muscle pain, concentration problems, and headache [2]. Cardiovascular complications are also common and include myocardial injury, myocarditis, acute myocardial infarction, heart failure, dysrhythmias, and venous thromboembolic events [3].

Among the post-COVID-19 sequelae, persistent pulmonary disease has recently gained attention because of the potential consequences in terms of morbidity and mortality [4]. Several studies investigated the respiratory outcomes after acute COVID-19, reporting different persisting lung abnormalities or functional impairment in up to 70% of patients at variable follow-up times [5–8]. More recently, a longer-term study from Wuhan reported that, in patients without significant comorbidities, radiological abnormalities were still detectable in 24% of them after 12 months of follow-up [9].

In this study, we aim to investigate long-term symptoms and respiratory outcomes over 12 months in a cohort of patients hospitalized for severe COVID-19.

## Patients and methods

### Study design and participants

In this prospective cohort study, consecutive adult patients with severe COVID-19 hospitalized and discharged from the Hospital of Fermo, Marche, Italy, were included in a longitudinal follow-up and evaluated every 6 months in a dedicated outpatient setting.

The inclusion criteria were as follows: diagnosis of severe COVID-19 based on the WHO interim guidance; laboratory confirmation of SARS-CoV-2 infection using real-time (RT)-PCR; hospitalization because of severe disease. Patients were defined to have severe COVID-19 if at least one of the following was present: respiratory rate ≥ 30 breaths/min; arterial oxygen saturation ≤ 93% at rest; PaO2/FiO2 ≤ 300 mmHg [10]. Patients were excluded if they were unwilling to be followed up at our Institution; if they were unable to sign the informed consent or comply with the study procedures.

Written informed consent was obtained from all study participants. This study was approved by the local Ethics Committee (CERM, 2021/249).

### Procedures

The patients were assessed at 6 months and 12 months after discharge. During the visit, they were interviewed and underwent physical examination, routine blood test, pulmonary function tests, chest high-resolution CT (HRCT) scan, and a standardized 6 min walking test (6MWT).

Anxiety and depression symptoms were evaluated by two self-report measures: Zung’s Self-Rating Depression Scale (SDS) and Self-Rating Anxiety Scale (SAS). While the gold standard for the diagnosis of mental disorders remains the structured clinical interview, self-report measures are frequently employed in research studies [11]. In case of normal range of SDS or SAS we scored 0, in case of mildly-moderately range for anxiety or depression we scored 1, in case of severe depression or extreme anxiety levels we scored 2.

6MWT was done according to the ATS practical guidelines [12]. Each patient walked on the flat ground as fast as possible without oxygen inhalation and completed the 6MWT test independently. The results were expressed as meters. Oxygen desaturation after 6MWT was also recorded.

Pulmonary function tests were done according to American Thoracic Society (ATS)-European Respiratory Society guidelines [13]. The following parameters were measured: diffusing capacity of the lungs for carbon monoxide (DLCO); forced expiratory flow between 25 and 75% of forced vital capacity (FEF25-75); functional residual capacity; FVC; FEV1; residual volume; total lung capacity; and vital capacity. DLCO was measured using the single-breath test. The hemoglobin value was taken for correcting the DLCO. For spirometry, flow-volume curves were obtained through a dry spirometer (Quark PFT, Cosmed, Italy) and the greatest volume of the three maneuvers was expressed as the percentage of predicted normal and used for analysis. All pulmonary function test measurements were expressed as percentages of predicted normal values. Diffusion deficit was considered as DLCO less than 75% of predicted values.

At 6 and 12 months, patients underwent chest non-contrast enhanced CT scan if they had still symptoms or detectable abnormalities at the last scan. CT was performed in the supine position and with breath-holding following inspiration when the patient was able to hold breath. The patients were imaged with 3 mm slice thickness CT on a 16 rows CT scanner (Philips Brilliance; Amsterdam, Netherlands). Images were obtained with both mediastinal (width 380 HU; level 60 HU) and parenchymal (width 1800 HU; level 500 HU) window settings. Two experienced radiologists independently reviewed the images, unaware of the clinical information or status of the patients. The % of lung involvement on CT was evaluated for all scans, as well as the following lung abnormalities: ground-glass attenuation, consolidation, interlobular septal thickening, reticular opacities, traction bronchiectasis, honeycombing, non-specific fibrotic changes, mosaic attenuation, air trapping, tree-in-bud alterations, centrilobular nodules.

### Statistical analysis

Continuous variables were expressed as median (1st–3rd quartiles) and compared using non-parametric tests (Mann–Whitney *U* test or Wilcoxon signed rank test), while categorical variables were expressed as *n* (%) and compared by chi square test or McNemar test. Correlations between variables was estimated using the Spearman’s rank correlation coefficient. Missing data were considered negligible if they were less than < 5%. *p* values less than 0.05 were considered statistically significant. All data analyses and graphs were done in STATA (v 14.0).

## Results

### Patients characteristics

**Fig. 1:**
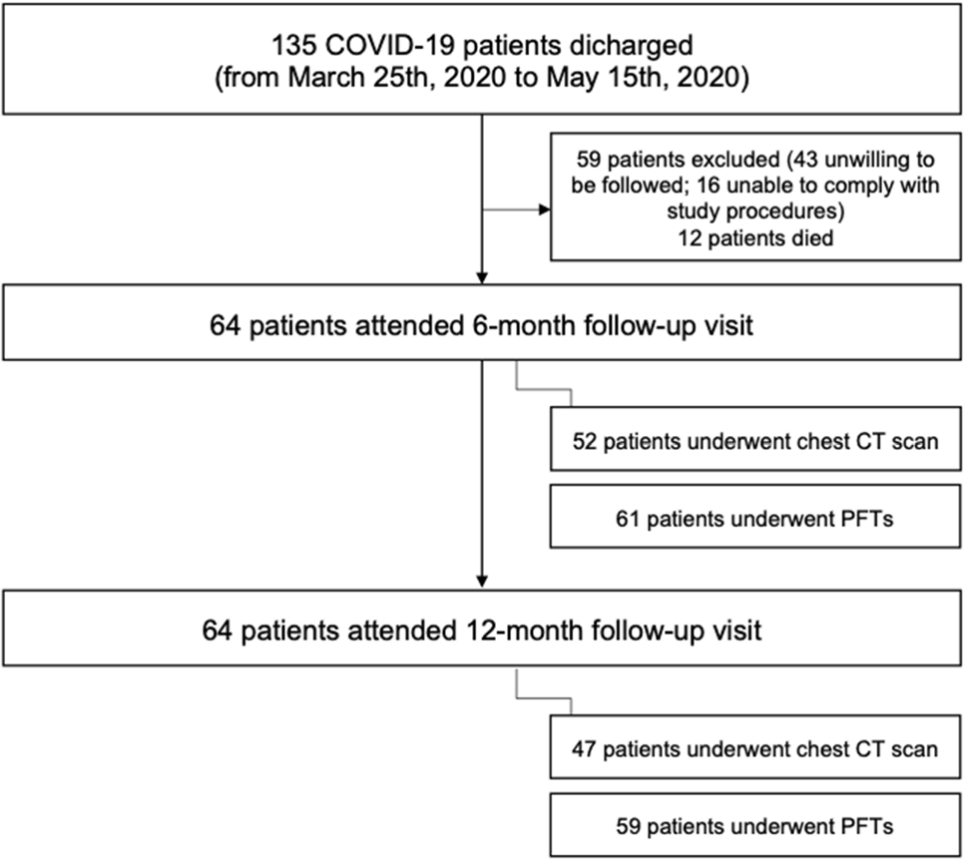
Flow-chart showing the selection of the study population

A total of 64 patients were eligible to be included in the study (Fig. 1, Table 1). All the patients had been discharged between March 25th, 2020 and May 15th, 2020. The majority of patients were male (64%), and the median age was 68 years. A considerable percentage of patients had comorbidities, including hypertension, obesity and diabetes. Forty percent of the patients were former smokers but only 3 patients were active smokers. The majority of patients (86%) needed oxygen supply during hospitalization, while about half of them required cycles of non-invasive ventilation.

**Table 1:** Baseline characteristics of Covid-19 patients included in the study

Variable		Total (*n* = 64)
Age, median (1st–3rd quartiles)		68 (56.5–75)
Male, *n* (%)		41 (64)
BMI (kg/m^2^), median (1st–3rd quartiles)		26 (24–27.8)
Comorbidities, *n* (%)		
	Hypertension	33 (51.5)
	Diabetes	12 (18.7)
	Obesity	8 (13)
	Heart disease	16 (25)
	Lung disease	11 (17)
	Active cancer	4 (6.2)
	Rheumatic diseases	6 (9.4)
	Current smokers	3 (4.6)
	Former smokers	25 (39)
Oxygen supply, *n* (%)		
	Orotracheal intubation	9 (14)
	Non-invasive ventilation	29 (45)
	All oxygen supply	55 (86)
Symptoms at baseline, *n* (%)		
	Dyspnea	54 (84)
	Cough	30 (47)
	Fatigue	35 (55)
	Diarrhea	13 (20)
	Joint pain	12 (18)
	Headache	3 (4.6)
	Attention and/or memory deficit	2 (3)
CT findings at baseline, *n* (%)		*n* = 60
	Ground glass	58 (96.7)
	Consolidation	52 (86.7)
	Interlobular thickening	51 (85)
	Reticular opacity	36 (60)
	Traction bronchiectasis	4 (6.7)
	Honeycombing	1 (1.7)
	Non-specific fibrotic changes	5 (8.3)
	Mosaic attenuation	/
	Air trapping	/
	Tree-in-bud	/
	Centrilobular nodules	/
	% of lung involvement, median (1st–3rd quartiles)	42.5 (35–60)

At baseline, most patients reported symptoms such as dyspnea (84%), cough (47%), fatigue (55%) and joint pain (18%). The most common HRCT findings at baseline were ground-glass opacity (96.7%), consolidation (86.7%), interlobular thickening (85%) and reticular opacity (60%).

### Outcomes at 6 months

At 6 months (Table 2), there was a significant reduction of symptoms such as dyspnea (36% vs 85%, *p* < 0.001), cough (5% vs 47%) and fatigue (37.5% vs 55%). More than 1 in 10 patients reported memory or attention deficits and more than 1 in 3 annoying hair loss. Importantly, anxiety and depression were reported by 48.5% and 56.4% of patients, respectively.

**Table 2:** Symptoms, PFTs and CT findings at 6 and 12 months

Variable		Total (*n* = 64)
Symptoms at 6 months, *n* (%)		
	Dyspnea	23 (36)
	Cough	3 (5)
	Fatigue	24 (37.5)
	Diarrhea	0 (0)
	Joint pain	19 (14)
	Headache	1 (1)
	Attention and/or memory deficit	7 (11)
	Hair loss	19 (30)
	Anosmia	2 (3)
	Anxiety	31 (48.5)
	Depression	36 (56.3)
Pulmonary function tests at 6 months, median (1st–3rd quartiles)		*n* = 61
	FVC	97 (83–108)
	FEV1	97 (86–112)
	FEF25-75	89 (74–11j5)
	TLC	96 (90–114)
	DLCO	73 (67–85)
	FVC reduced, *n* (%)	13 (21.3)
	DLCO reduced, *n* (%)	35 (57.4)
CT findings at 6 months, *n* (%)		*n* = 52
	Ground glass	22 (42.3)
	Consolidation	10 (19)
	Interlobular thickening	17 (33)
	Reticular opacity	26 (50)
	Traction bronchiectasis	4 (8)
	Honeycombing	3 (5.7)
	Non-specific fibrotic changes	8 (15.4)
	Mosaic attenuation	3 (5.7)
	Air trapping	/
	Tree-in-bud	/
	Centrilobular nodules	/
	% of patients with abnormal findings, *n* (%)	42 (80.7)
	% of lung involvement, median (1st–3rd quartiles)	10 (5–25)
Symptoms at 12 months, *n* (%)		
	Dyspnea	12 (18.7)
	Cough	4 (6.2)
	Fatigue	8 (12.5)
	Diarrhea	0 (0)
	Joint pain	4 (6.2)
	Headache	1 (1)
	Attention and/or memory deficit	3 (4.7)
	Hair loss	1 (1.5)
	Anosmia	1 (1)
	Anxiety	32 (50)
	Depression	39 (61)
Pulmonary function tests at 12 months, median (1st–3rd quartiles)		*n* = 59
	FVC	99 (89–112)
	FEV1	99 (90–115)
	FEF25-75	87 (61–109)
	TLC	97.5 (87.5–112.5)
	DLCO	77 (64–82)
	FVC reduced, *n* (%)	9 (14.7)
	DLCO reduced, *n* (%)	28 (47.7)
CT findings at 12 months, *n* (%)		*n* = 47
	Ground glass	7 (15)
	Consolidation	7 (15)
	Interlobular thickening	5 (10.6)
	Reticular opacity	19 (40.4)
	Traction bronchiectasis	4 (8.5)
	Honeycombing	2 (4.2)
	Non-specific fibrotic changes	7 (15)
	Mosaic attenuation	1 (2)
	Air trapping	/
	Tree-in-bud	1 (2)
	Centrilobular nodules	/
	% of patients with abnormal findings, *n* (%)	30 (63.8)
	% of lung involvement, median (1st–3rd quartiles)	5 (0–10)

Pulmonary function tests were within normal ranges for most patients. However, 21.3% of patients had reduced FVC and 57.4% met the cut-off for low DLCO.

Of the 52 patients for which a chest CT scan was available, 80.7% showed signs of any abnormality, although there was a reduction of the % of lung involvement (10% vs 42.5%). The most common patterns were ground glass (42.3%), reticular opacity (50%), interlobular thickening (33%), consolidation (19%) and non-specific fibrotic changes (15.4%).

### Outcomes at 12 months

Persisting symptoms were also reported at 12 months (Table 2), most commonly dyspnea (18.7%), cough (6.2%), fatigue (12.5%), joint pain (6.2%) and attention or memory deficit (4.7%). The percentage of patients that reported anxiety and depression increased to 50% and 61%, respectively.

**Fig. 2:**
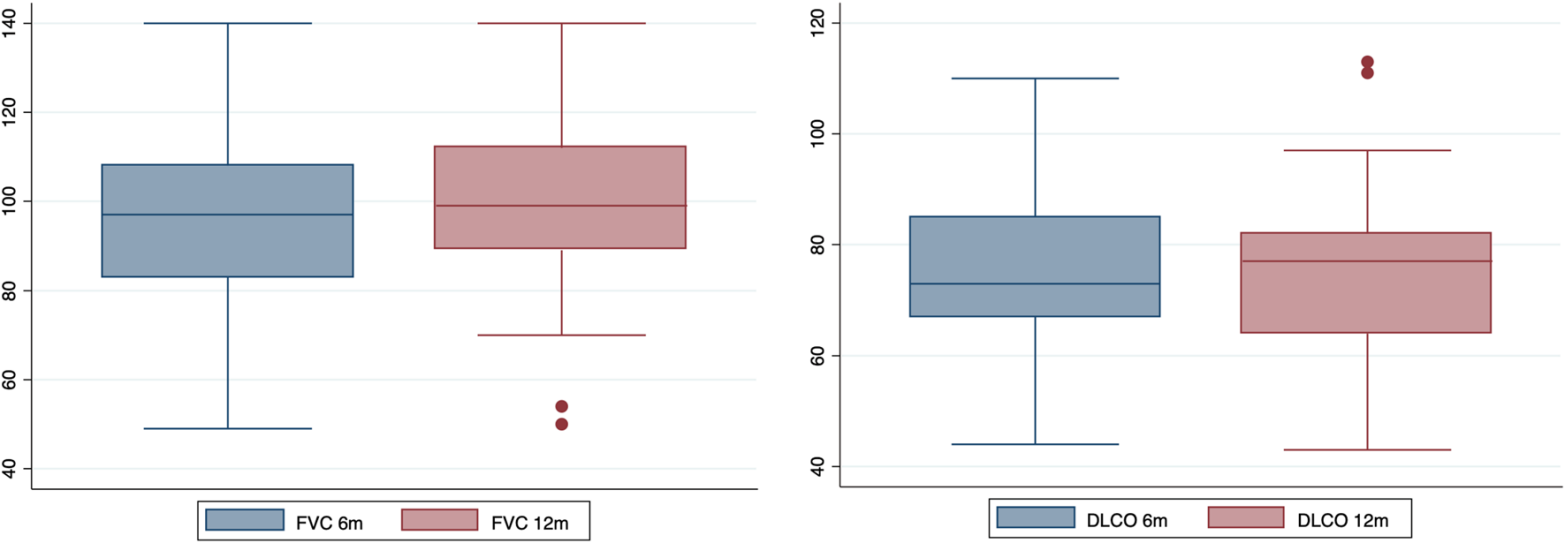
Box-plot graphs showing the improvement of FVC% and the stability of DLCO% between the 6 and 12 month follow-up

Compared to the 6 month assessment, the FVC% significantly increased (99 (89–112) vs 97 (83–108), *p* = 0.002), while TLC (97.5 (87.5–112.5) vs 96 (90–114), *p* = *ns*) and DLCO (77 (64–82) vs 73 (67–85), *p* = *ns*) remained stable (Fig. 2). Almost half of the patients had reduced DLCO at 12 months.

Lung abnormalities were still detectable in 63% of the 47 patients with a chest CT scan at 12 months, tough with a low % of lung involvement (5% (0–10)). The most common patterns were still ground glass (15%), reticular opacity (40.4%), interlobular thickening (10.6%), consolidation (15%) and non-specific fibrotic changes (15%).

**Fig. 3:**
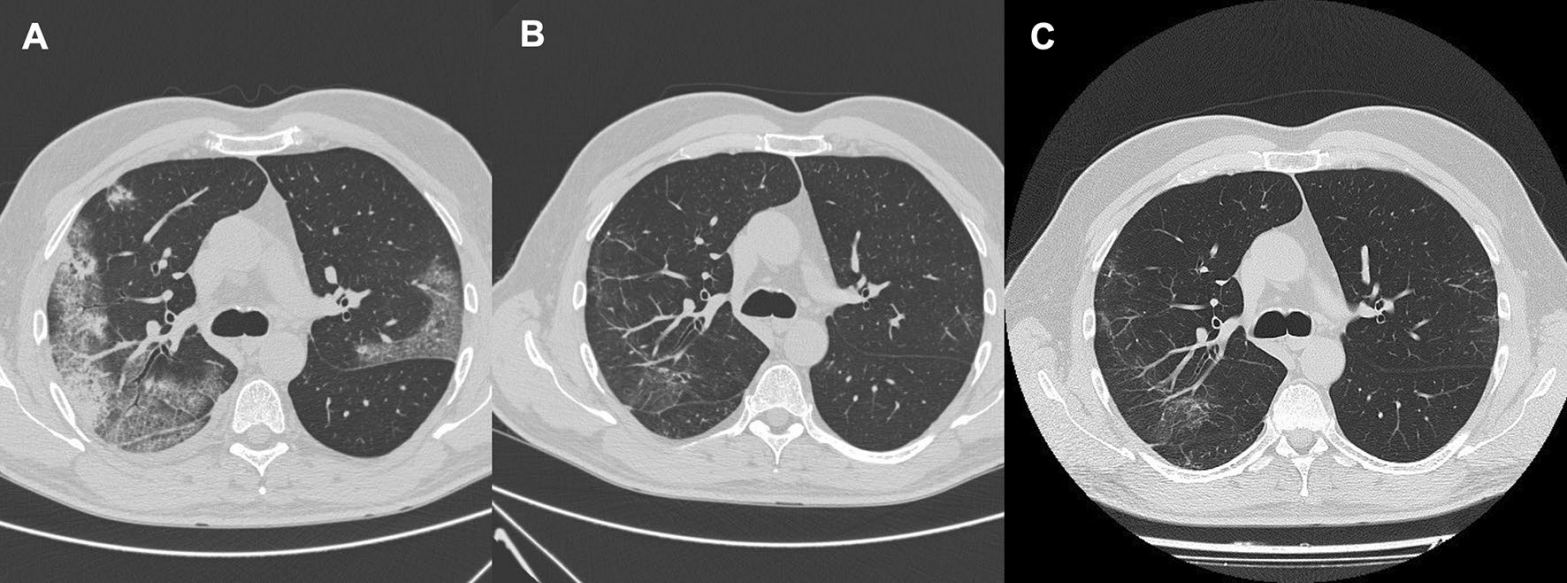
**A** Axial CT showing bilateral ground-glass and consolidative opacities and interlobular septal thickening. **B** At 6 months there is a reduction of ground-glass and consolidative opacities and of interlobular septal thickening. **C** At 12 months there is a resolution of ground-glass and consolidative opacities and of interlobular septal thickening and the appearance of subpleural curvilinear opacities

The improving trend of lung involvement on HRCT across 12 months is shown in Fig. 3. In this patient, there was a reduction and almost complete resolution of ground-glass and consolidative opacities and of interlobular septal thickening.

### Correlation between functional and radiological abnormalities

We next compared patients with normal vs abnormal DLCO at 12 months (Table 3) and found that patients with reduced DLCO were older (72.5 vs 60 years, *p* = 0.01), more frequently reported dyspnea (25% vs 6.5%, *p* = 0.04) and had reduced FEV1 (94 (86–102) vs 105 (93–117), *p* = 0.02) and TLC (93.5 (87.5–105) vs 105 (89–121), *p* = 0.02). More importantly, a higher proportion of patients with reduced DLCO showed any lung abnormality on CT (81% vs 47.6%, *p* = 0.02) with a higher % of lung involvement (15% vs 5%, *p* = 0.04).

**Table 3:** Comparison between patients with normal/abnormal DLCO at 12 months (*n* = 59)

Variable		Normal DLCO (*n* = 31)	Abnormal DLCO (*n* = 28)	*p*
Age, median (1st–3rd quartiles)		60 (53–70)	72.5 (65.5–78.5)	0.01
Male, *n* (%)		21 (67.7)	17 (60.7)	0.57
BMI (kg/m^2^), median (1st–3rd quartiles)		26 (24–28.5)	26 (24–27.5)	0.82
Smoking habit, *n* (%)		15 (48.5)	10 (25.7)	0.30
Symptoms, *n* (%)				
	Dyspnea	2 (6.5)	7 (25)	0.04
	Cough	1 (3.2)	3 (10.7)	0.25
	Fatigue	2 (6.5)	5 (17.8)	0.17
	Anxiety	15 (48.4)	14 (50)	0.90
	Depression	17 (54.8)	19 (67.8)	0.37
6MWT, median (1st–3rd quartiles)		517.5 (460–580)	461.5 (380–536)	0.12
Desaturation at 6MWT, *n* (%)		4 (14.3)	2 (7.7)	0.44
PFTs, median (1st–3rd quartiles)				
	FVC	105 (93–117)	95 (88–104)	0.06
	FEV1	105 (93–117)	94 (86–102)	0.02
	FEF25-75	104 (68–114)	77 (60–98.5)	0.07
	TLC	105 (89–121)	93.5 (87.5–105)	0.02
CT findings, *n* (%) (*n* = 47)				
	Ground glass	2 (9.5)	4 (19)	0.37
	Consolidation	2 (9.5)	4 (19)	0.37
	Interlobular thickening	1 (4.7)	3 (14.2)	0.29
	Reticular opacity	7 (33)	10 (47.6)	0.34
	Traction bronchiectasis	3 (14.3)	1 (4.7)	0.29
	Non-specific fibrotic changes	3 (14.3)	4 (19)	0.67
	Patients with abnormal findings, *n* (%)	10 (47.6)	17 (81)	0.02
	% of lung involvement, median (1st–3rd quartiles)	5 (0–20)	15 (5–25)	0.04

Table 4 shows the comparison between patients with abnormal CT findings at 12 months. There were no differences between the two groups with regard to age, sex, smoking habit, symptoms, oxygen saturation on 6MWT and most PFTs parameters, except for DLCO, that was significantly lower in patients with lung abnormalities (70 (62–78) vs 79 (70–93), *p* = 0.01).

**Table 4:** Comparison between patients with normal/abnormal CT at 12 months (*n* = 47)

Variable		Normal CT (*n* = 31)	Abnormal CT (*n* = 28)	*p*
Age, median (1st–3rd quartiles)		60 (56–72)	71.5 (66–78)	0.06
Male, *n* (%)		13 (76.4)	18 (60)	0.25
BMI (kg/m^2^), median (1st–3rd quartiles)		25.8 (24.3–28.6)	25.6 (23.5–27)	0.44
Smoking habit, *n* (%)		10 (58.7)	10 (33.3)	0.23
Symptoms, *n* (%)				
	Dyspnea	3 (17.6)	7 (23.3)	0.64
	Cough	0 (0)	3 (10)	0.17
	Fatigue	1 (5.8)	6 (20)	0.19
	Anxiety	11 (64.7)	14 (46.6)	0.23
	Depression	13 (76.4)	18 (60)	0.39
6MWT, median (1st–3rd quartiles)		377 (368–395)	353.5 (339–387.5)	0.52
Desaturation at 6MWT, *n* (%)		2 (14.3)	5 (18.5)	0.73
PFTs, median (1st–3rd quartiles)				
	FVC	94 (86.5–113)	99 (88–108)	0.97
	FEV1	99 (91.5–117.5)	97.5 (86–110)	0.51
	FEF25-75	103.5 (73–114.5)	75 (51.5–110)	0.11
	TLC	101 (92–108)	93.5 (82.5–108)	0.22
	DLCO	79 (70–93)	70 (62–78)	0.01
	Reduced DLCO, *n* (%)	4 (26.6)	17 (63)	0.02

We could not find any influence of biological sex on symptoms, PFTs parameters and HRCT abnormalities at all time points (data not shown), except for a marginally significant difference in the rate of anxiety (65.2% F vs 39% M, *p* = 0.04) at 6 months, that was not confirmed at 12 months.

Finally, the results of the Spearman analysis showed that FVC and DLCO were weakly but significantly correlated (rho 0.36, *p* = 0.004), as were TLC and DLCO (rho 0.46, *p* = 0.0002). DLCO was also weakly correlated to the % of lung involvement (rho −0.31, *p* = 0.04) and to the distance walked at the 6MWT (rho 0.27, *p* = 0.04).

## Discussion

Several studies have shown that long Covid can affect the whole spectrum of people with COVID-19, from those with very mild acute disease to the most severe forms. Like acute COVID-19, post-acute COVID-19 syndrome can involve multiple organs and systems including, but not limited to, the respiratory, cardiovascular, neurological, gastrointestinal, and musculoskeletal systems [1].

In our work, we reported the 12 month outcomes of patients hospitalized for acute COVID-19, showing that clinical symptoms and radiographic changes may persist in part of the patients.

In particular, at 12 months clinical symptoms were still reported by up to one in five patients. Importantly, while the most common symptoms such as dyspnea and fatigue improved throughout the follow-up, depression and anxiety were reported by at least half of the patients after 1 year. This prompts the need for an accurate screening of psychological well-being of COVID-19 survivors, and further reinforces the impact of this pandemic in the mental health of the population [14].

As in previous studies, we could demonstrate that low DLCO is the most common functional abnormality in COVID-19 survivors, especially in those with more severe or critical disease [5], 8]. However, while residual abnormalities of pulmonary function, as well radiographic changes, were found in up to half of the patients, we failed to demonstrate a strong correlation with reduced exercise capacity or oxygen desaturation at 6MWT. If on one hand patients with reduced DLCO at 12 months had more commonly detectable radiographic changes and more frequently reported persistent dyspnea than those with DLCO in the normal range, on the other hand we found no differences in exercise capacity in patients with normal or reduced DLCO, as well as in those with or without any residual chest CT abnormality at 12 months, despite the weak correlation between DLCO and distance walked at the 6MWT.

The most common radiographic abnormalities reported at 6 and 12 months were ground glass and reticular opacities. Despite the relatively high rate of patients with any abnormalities, a minority of lung parenchyma was generally involved and, more importantly, the abnormalities were not suggestive of a fibrotic interstitial lung disease phenotype.

In one of the first studies reporting outcomes at 1 year [9], albeit conducted in patients without significant comorbidities, HRCT was abnormal in one in four patients and DLCO was reduced in one in three patients at 12 months. Importantly, despite significant reduction of most PFTs parameters in patients with radiological changes, none of the HRCT scans showed evidence of established or progressive fibrosis. In addition, the time course of symptoms, exercise capacity, functional and radiological changes showed a significant improvement from 3 to 12 months. In the largest study of long-term sequelae so far [15], the Authors report a 50% prevalence of any symptom at 12 months; moreover, one in four patients had either anxiety or depression. In the subset of patients for which functional and radiographic data were available, FVC% and HRCT abnormalities improved between 6 and 12 months, but not DLCO, remaining decreased in 23–54% of them at 12 months.

These results are consistent with our findings, suggesting that pulmonary sequelae of COVID-19 could be transient and mostly short-lived. Indeed, to establish the clinical significance of DLCO reduction in this context further investigation is needed. Whereas in previous studies DLCO reduction has been associated with female sex and higher disease severity, in the study by Wu et al. [9] women did not show a higher percentage of HRCT abnormalities despite having a lower DLCO. Although we cannot draw firm conclusions due to the small sample size, we have not observed a significant effect of biological sex on disease outcomes either. Thus, while reduced DLCO is thought to be the consequence of interstitial abnormalities or pulmonary vascular abnormalities caused by COVID-19, other mechanisms may underlie the persistent long-term impairment of gas-blood exchange. Finally, other Authors reported a high prevalence of air trapping and a very low prevalence of interstitial abnormalities in their cohort of patients with persisting respiratory symptoms [16], but we did not observe abnormalities suggestive of small airways disease in our cohort.

Compared to previous reports, the strengths and novelty of our study consisted of long-term follow-up and the inclusion of a broad spectrum of patient severity, as we included unselected patients with various comorbidities and also those who required mechanical ventilation.

There are also several limitations in this study. First of all, the small sample size and the selection of the patients may hamper the generalizability of study results. Second, the inclusion of patients with severe disease may overestimate post-acute sequelae or ignore other problems in patients with milder COVID-19. Third, we did not have any information about the functional and radiological status of the patients before COVID-19, as well as their previous psychological status.

## Conclusion

In conclusion, persistent symptoms and pulmonary abnormalities are common after 1 year in patients admitted for COVID-19. Depression and anxiety have been reported by at least half of patients and we have found a persistent reduction in DLCO in many patients. A comforting finding is that HRCT scans showed no evidence of established or progressive fibrosis. An extended follow-up is warranted to assess the temporal trends, the best management and the prognostic relevance of the long-term consequences of severe COVID-19.
